# Ultrasound-Assisted Deep Eutectic Solvent Extraction of Total Alkaloids from *Eupatorium lindleyanum*: Process Optimization and Preservation Application in Postharvest Grapes

**DOI:** 10.3390/molecules31183352

**Published:** 2026-09-21

**Authors:** Zhimeng Xu, Hanbing Yuan, Ling Li, Yanwen Yu, Menghan Guo, Yanming Wang, Jun Yuan

**Affiliations:** 1 Jiangsu Key Laboratory of Regional Specific Resource Pharmaceutical Transformation, Huai’an University, Huai’an 223003, China; 12021004@hau.edu.cn (Z.X.); 212406560139@hau.edu.cn (H.Y.); 212501600205@hau.edu.cn (L.L.); 212601600205@hau.edu.cn (Y.Y.); 112506040107@hau.edu.cn (M.G.); wym@hau.edu.cn (Y.W.); 2 National & Local Joint Engineering Research Center for Mineral Salt Deep Utilization, Huai’an University, Huai’an 223003, China

**Keywords:** *Eupatorium lindleyanum*, deep eutectic solvents, alkaloids, ultrasound-assisted extraction, postharvest grape preservation

## Abstract

A green efficient deep eutectic solvent (DES)-based system was developed for total alkaloid extraction from *Eupatorium lindleyanum* DC. and evaluated for postharvest grape preservation. Among 18 DESs, choline chloride–citric acid (2:1, molar ratio) showed the highest extraction efficiency. Box–Behnken design and response surface methodology optimized the process to 52 min, 42 °C, and a liquid-to-solid ratio of 39:1 mL∙g^−1^, yielding 277.96 mg∙g^−1^ total alkaloids. SEM showed pronounced structural disruption of *E. lindleyanum* tissues after UAE-DES treatment. The DES extract exhibited DPPH and ABTS radical scavenging activities of 72.74% and 77.35%, respectively, significantly higher than those of the water extract. In postharvest grapes, the DES extract reduced MDA accumulation and maintained higher VC levels. In *Botrytis cinerea*-inoculated grapes, it reduced visible gray mold-associated deterioration and alleviated oxidative damage. This study extends UAE-DES research from extraction optimization to evaluation in a real postharvest fruit system and supports the potential of *E. lindleyanum* extracts with high total alkaloid content for natural antioxidant-based preservation.

## 1. Introduction

Alkaloids are a class of nitrogen-containing secondary metabolites widely distributed in medicinal plants and well known for their diverse biological activities, including antioxidant, antibacterial, antifungal, anti-inflammatory, and cytotoxic properties [1]. Owing to their antioxidant and antimicrobial activities, alkaloids have attracted increasing attention as potential natural preservatives for food preservation and postharvest quality maintenance. Mechanistically, the antioxidant effects of alkaloids may involve the scavenging of reactive oxygen species (ROS), thereby reducing oxidative damage associated with postharvest ripening and senescence [2]. In addition, some alkaloids can exert antimicrobial effects by increasing bacterial membrane permeability and interfering with essential cellular processes, including enzyme activity and cell division, thereby inhibiting microbial growth [3]. Together, these antioxidant and antimicrobial actions may help delay oxidative deterioration and microbial spoilage during postharvest storage. Previous studies have shown that berberine exhibits antibacterial activity against several food-related microorganisms, including *Escherichia coli*, *Staphylococcus aureus*, and *Aspergillus* species [4]. In addition, tomatine from tomatoes exhibits antimicrobial and antifungal activities, supporting its potential application in food preservation [5]. Capsaicin from chili peppers also exhibits strong antimicrobial and antioxidant activities, suggesting its potential as a natural food preservative and additive [6]. These studies highlight the considerable potential of plant-derived alkaloids in food preservation and postharvest quality maintenance. However, their practical application remains limited by the low efficiency of conventional extraction methods, high solvent consumption, and insufficient validation in real food systems. Therefore, green and efficient extraction technologies are needed to improve alkaloid recovery and support their practical application in food preservation.

*Eupatorium lindleyanum* DC. is an edible medicinal herb widely distributed in China and has long been used in traditional medicine for the treatment of respiratory and inflammatory diseases. Phytochemical studies have revealed that *E. lindleyanum* contains various bioactive constituents, including alkaloids, flavonoids, sesquiterpenes, and polysaccharides, among which alkaloids are considered one of the major active components. Previous studies on *E. lindleyanum* have employed conventional solvent-based extraction approaches. For example, Ji et al. used water decoction and reflux extraction with ethyl acetate, 75% ethanol, and methanol [7]. For the organic-solvent extractions, *E. lindleyanum* powder was refluxed at 60 °C for 10 h, followed by repeated reflux extraction of the residues. The crude extract recoveries obtained using 75% ethanol, water decoction, methanol, and ethyl acetate were 17.6, 15.3, 11.3, and 5.0 g per 100 g of dry material, respectively [7]. Organic-solvent-based extraction has also been employed for the analysis of *E. lindleyanum* alkaloids [8]. These conventional procedures may require prolonged extraction, repeated processing steps, and relatively large amounts of solvent, thereby increasing processing time, solvent consumption, and energy demand. Prolonged thermal exposure may also increase the risk of degradation of thermally sensitive constituents. A recent food microbiology study demonstrated that *E. lindleyanum* extract inhibited eight common food spoilage and foodborne pathogenic bacteria, including *Bacillus subtilis*, *Listeria monocytogenes*, and *Salmonella typhimurium*, with minimum inhibitory concentration (MIC) values ≤ 4 mg∙mL^−1^, highlighting its potential as a natural preservative for postharvest fruits and ready-to-drink beverages [7]. Moreover, *E. lindleyanum* extract has also shown promising potential in the development of compound natural preservatives for chilled meat products, further supporting its application in food preservation. Taken together, the relatively long extraction time, repeated extraction steps, solvent consumption, and thermal exposure associated with conventional methods highlight the need for a green, efficient, and sustainable extraction strategy for *E. lindleyanum* alkaloid recovery.

Deep eutectic solvents (DESs), formed by mixing hydrogen bond donors (HBDs) and hydrogen bond acceptors (HBAs) at specific molar ratios, have emerged as a new generation of green solvents owing to their low volatility, tunable physicochemical properties, and environmental compatibility [9,10,11,12,13]. For alkaloid extraction, the tunable polarity of DESs and their ability to establish hydrogen-bonding interactions with alkaloid molecules can facilitate the solubilization of target compounds, while their compatibility with ultrasound can further enhance solvent penetration and mass transfer [13,14]. When combined with ultrasound-assisted extraction (UAE), DESs can enhance mass transfer, promote cell wall disruption, and facilitate the release of intracellular compounds under mild conditions [15,16,17]. In recent years, DES-based extraction systems, including UAE-DES, have been successfully applied to the extraction of alkaloids from various medicinal plants. DES-based extraction has been applied to alkaloid recovery from *Euchresta tubulosa Dunn* [18], *Phellodendri amurensis* cortex [14], and *Evodia lepta* [13]. These studies indicate that DES-assisted extraction provides an effective and sustainable strategy for improving alkaloid recovery from plant materials. In contrast to previous studies focusing primarily on alkaloid extraction from other plant matrices, the present study applies UAE-DES to total alkaloids from *E. lindleyanum* and further evaluates the antioxidant activity and preservation performance of the obtained extract in a real postharvest grape system. Thus, the present work links extraction-process optimization with practical food preservation evaluation, extending the application scope of UAE-DES beyond extraction alone.

In this study, a UAE-DES extraction system was developed for the extraction of total alkaloids from *E. lindleyanum*. The extraction process was optimized using single-factor experiments and response surface methodology (RSM). The antioxidant activity of the obtained extracts was evaluated, and their preservation performance in postharvest grapes was further investigated.

## 2. Results and Discussion

### 2.1. DES Screening

**Figure 1:**
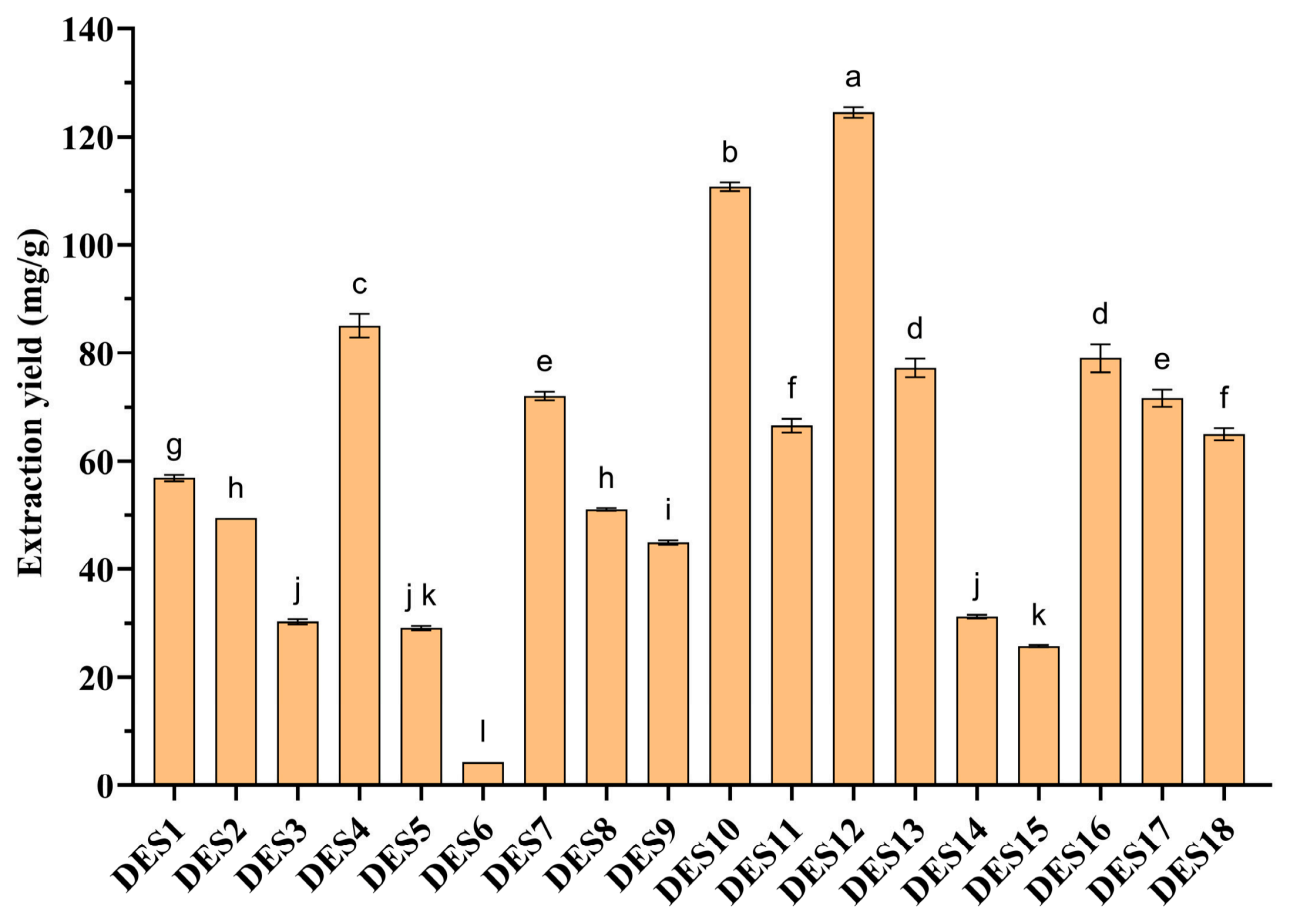
TA of *E. lindleyanum* extracts obtained using 18 DESs. Data are expressed as mean ± SD (*n* = 3). Groups sharing at least one lowercase letter are not significantly different, whereas groups with no letters in common are significantly different according to one-way ANOVA followed by Tukey’s multiple comparison test (*p* < 0.05).

According to the major HBA/HBD components, the prepared DESs were classified into five representative groups. For DESs containing both organic acids and sugars, the organic acid component was used as the primary classification criterion. These groups included organic acid-containing DESs (DES1, DES2, DES4, DES7, DES10, DES12, and DES13), sugar/sugar alcohol-containing DESs (DES3, DES6, DES8, and DES9), polyol-based DESs (DES5, DES14, and DES15), amide-based DES (DES11), and hydrophobic DESs (DES16–DES18). Eighteen DESs covering these different HBA/HBD classes were evaluated under fixed UAE conditions (50 °C, 50 min, and a liquid-to-solid ratio of 20 mL∙g^−1^). As shown in Figure 1, ChCl–citric acid (2:1, molar ratio; DES12) exhibited the highest total alkaloid (TA) yield of 124.5 ± 1.0 mg∙g^−1^, which was 28.8-fold higher than that obtained using Bet–glucose (DES6, 4.32 mg∙g^−1^).

The composition of DESs can markedly influence their physicochemical properties, such as polarity and hydrogen-bonding capability, thereby affecting the extraction performance of target bioactive compounds [19]. To elucidate the reasons underlying the differences in the extraction performance of different DESs, the density and polarity of representative DESs were determined. Four representative hydrophilic DESs (DES1, DES6, DES7, and DES12) with different HBA/HBD combinations and distinct extraction performances were selected for physicochemical characterization, and the results are presented in Table 1. Among them, DES6 showed the lowest TA yield, whereas DES12 exhibited the highest yield. DES1 and DES7 were selected as additional hydrophilic DESs containing sugar and/or organic acid components, allowing further comparison of how solvent composition affected density, polarity, and extraction performance. The densities of DES1, DES6, DES7, and DES12 ranged from 1.2999 to 1.3384 g∙cm^−3^, indicating that all DESs formed relatively dense solvent networks through intermolecular interactions. Pearson correlation analysis showed no significant linear correlation between density and TA yield (Pearson’s *r* = 0.405, *p* = 0.595; Figure S1A), suggesting that density alone was unlikely to be the dominant factor governing alkaloid extraction. According to the principle of “like dissolves like”, solvents with polarity values close to those of the target compounds generally exhibit superior dissolution capability. Previous studies have also demonstrated that solvent polarity is one of the key factors governing the extraction of phytochemicals by regulating solvent–solute interactions and solvation capacity [20]. As shown in Table 1, the E_NR_ values of DES1, DES6, DES7, and DES12 were 44.6, 58.5, 47.7, and 44.7, respectively. Pearson correlation analysis showed a negative but non-significant correlation between ENR and TA yield (Pearson’s *r* = −0.826, *p* = 0.174; Figure S1B). DES12 exhibited a relatively moderate polarity, which was markedly lower than that of DES6 and comparable to that of DES1. The moderate polarity of DES12 may have contributed to the overall extraction of alkaloid-related constituents detected by the colorimetric assay. However, because individual alkaloids were not chromatographically identified in the present study, the contribution of specific alkaloid classes cannot be established. The similar polarity values of DES1 and DES12, despite their different extraction performances, indicate that polarity alone cannot fully explain the differences in extraction efficiency. In addition to polarity, viscosity may also affect DES extraction performance by influencing solvent penetration and mass transfer within the plant matrix. However, viscosity was not experimentally evaluated in the present study; therefore, its specific contribution to the superior extraction performance of DES12 remains to be clarified.

In addition to polarity, the nature of the hydrogen-bond donor may also affect the extraction performance. Compared with DES6, a sugar-containing DES with the lowest TA yield, the organic acid-containing DES12 exhibited markedly superior extraction performance. Previous studies have reported that choline chloride–organic acid DESs generally exhibit acidic characteristics [21], and that active protons in acidic DES systems can promote fiber swelling and improve matrix accessibility, which may facilitate mass transfer during extraction [22]. However, because the pH values of the DESs were not experimentally measured in the present study, the specific contribution of acidity to the enhanced extraction performance of DES12 cannot be directly established. Moreover, the choline chloride–citric acid system possesses strong hydrogen-bonding capability, which may enhance solvent–solute interactions and thereby facilitate alkaloid solvation and extraction [14]. Therefore, considering both its overall extraction performance and favorable hydrogen-bonding characteristics, DES12 was selected as the optimal solvent for subsequent extraction experiments.

### 2.2. Effects of Single-Factor Parameters on Total Alkaloid Yield

To determine the appropriate ranges of extraction time, temperature, and liquid-to-solid ratio, single-factor experiments were conducted using the optimal DES12 system, with the TA yield as the evaluation index.

#### 2.2.1. Extraction Time

**Figure 2:**
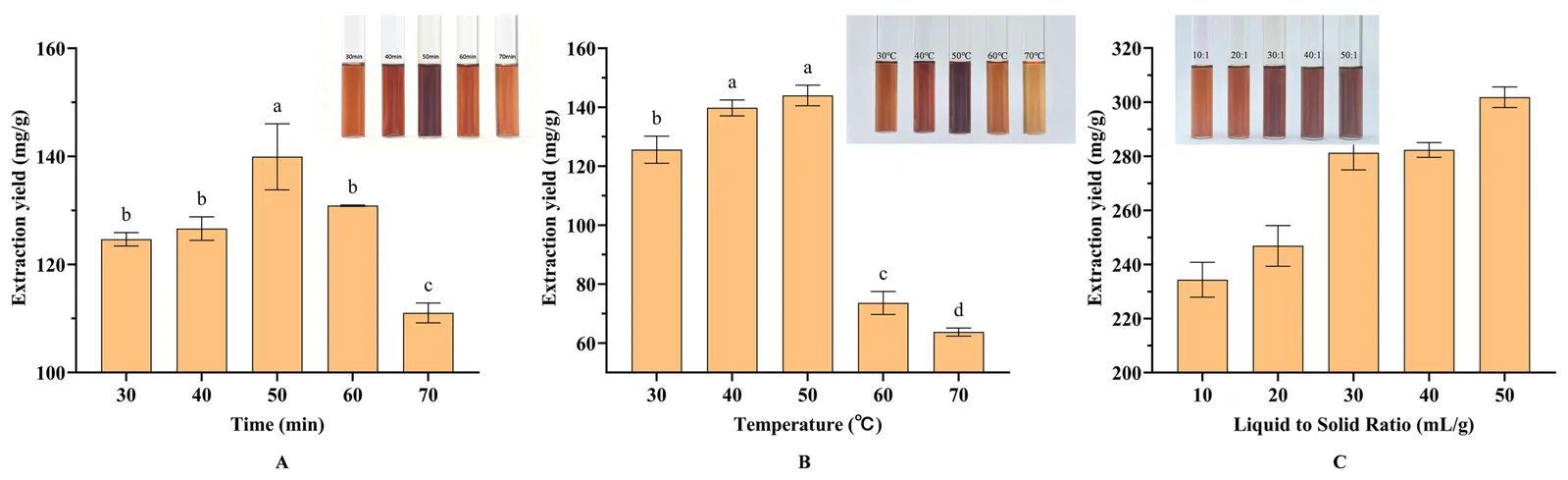
Effects of single-factor parameters on TA yield. (**A**) Extraction time. (**B**) Extraction temperature. (**C**) Liquid-to-solid (L/S) ratio. Data are expressed as mean ± SD (*n* = 3). Within each panel, groups sharing at least one lowercase letter are not significantly different, whereas groups with no letters in common are significantly different according to one-way ANOVA followed by Tukey’s multiple comparison test (*p* < 0.05).

According to the experimental procedure outlined in Section 3.6, the effect of extraction time (30–70 min) on the extraction efficiency of total alkaloids from *E. lindleyanum* was evaluated. As illustrated in Figure 2A, the total alkaloid content exhibited an initial increase followed by a decrease with prolonged extraction time, reaching a maximum at 50 min. This trend can be explained by the fact that a certain duration is required for efficient alkaloid extraction from the plant matrix; during the initial stage of extraction, ultrasound-induced cavitation and mechanical effects can disrupt the plant matrix, enhance solvent penetration, and promote the mass transfer of intracellular compounds into the extraction medium [23]. As extraction proceeds, the driving force for mass transfer gradually decreases as the extraction system approaches equilibrium, resulting in a reduced increase in extraction yield [23]. Insufficient time may leave significant amounts of alkaloids within the *E. lindleyanum* powder, whereas excessively long extraction times may cause thermal or chemical degradation of the target compounds [24]. Prolonged ultrasonication may also increase the risk of degradation or oxidation of extracted compounds [23]. Therefore, the extraction times of 40, 50, and 60 min were chosen as the three levels of RSM.

#### 2.2.2. Extraction Temperature

Following the test method of 3.6, the effects of extraction temperature (30–70 °C) on the total alkaloid extraction efficiency in *E. lindleyanum* were investigated. Figure 2B shows that with the increase in temperature, the total alkaloid content continuously increased and then gradually decreased, reaching the maximum at 50 °C. The trend indicates that appropriately increasing the operating temperature can enhance the solubility of alkaloids and improve the extraction efficiency. In addition, increasing temperature can reduce the viscosity of the extraction solvent and increase molecular diffusivity, thereby facilitating solvent penetration and mass transfer. Elevated temperature may also lower surface tension and weaken the interactions between target compounds and the plant matrix, promoting the desorption and dissolution of alkaloids into the extraction medium [25]. However, excessively high temperatures may adversely affect the stability of thermally sensitive alkaloids and promote their thermal degradation, resulting in a decrease in the total extracted alkaloid content [26]. Therefore, extraction temperatures of 40, 50, and 60 °C were chosen as the three levels of the RSM.

#### 2.2.3. Liquid-to-Solid (L/S) Ratio

Following the test method of 3.6, the effects of liquid-to-solid ratios (L/S ratios) of 10:1–50:1 (mL∙g^−1^) on the total alkaloid extraction efficiency in *E. lindleyanum* were investigated. Figure 2C shows that when the L/S ratio is low, the amount of solvent is too small, and the alkaloids in *E. lindleyanum* cannot be fully dissolved in the solvent. Increasing the L/S ratio provides a larger solvent volume and improves contact between the solvent and plant matrix, thereby maintaining a greater concentration gradient and facilitating solute diffusion and mass transfer [27]. However, an excessive amount of solvent will not only cause waste but may also extract other impurities. Impurities hinder the dissolution of alkaloids and lead to a decrease in extraction efficiency [28]. Once sufficient solvent is available for effective solubilization and mass transfer, further increases in the L/S ratio generally provide only limited improvement in extraction yield. From Figure 2C, it was obvious that when the L/S ratio is greater than 30:1 (mL∙g^−1^), the total alkaloid content increased slowly. To save resources, 20:1, 30:1, and 40:1 (mL∙g^−1^) were chosen as the three levels of the L/S ratio for the RSM experiments.

### 2.3. Optimization of Extraction Process with RSM

**Figure 3:**
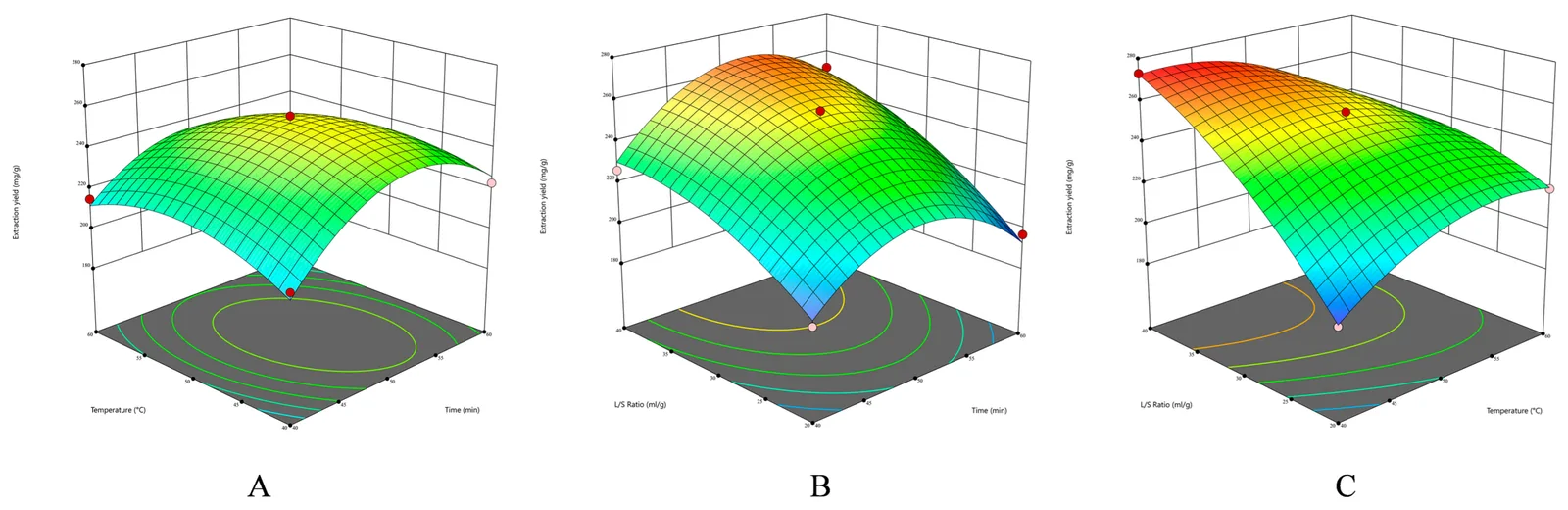
Response surface plots showing the interaction effects of the three factors on total alkaloid extraction yield. (**A**) Response surface plot for the temperature-time interaction. (**B**) Response surface plot for the L/S ratio-time interaction. (**C**) Response surface plot for the L/S ratio-temperature interaction.

Based on the results of the single-factor experiments, time (*A*, 40, 50, 60 min), temperature (*B*, 40, 50, 60 °C), and L/S ratio (*C*, 20:1, 30:1, 40:1 mL∙g^−1^) were selected as independent variables, and a regression equation was established with the total alkaloid extraction yield as the response value. The equation was then analyzed and validated. The Box–Behnken experimental design was employed, with a total of 15 experimental runs for three factors at three levels, and the design results are shown in Table 2 and Figure 3.

A multiple regression fitting analysis was performed on the experimental data in Table 2, and a quadratic polynomial regression model equation for the total alkaloid extraction yield (*Y*) was obtained: *Y* = 252.34 + 4.56 × *A* − 2.76 × *B* + 23.29 × *C* − 6.31 × *A* × *B* + 4.89 × *A* × *C* − 17.03 × *B* × *C* − 26.24 × *A*^2^ − 13.36 × *B*^2^ − 9.72 × *C*^2^.

Table 3 shows that the regression model was highly significant (*p* = 0.0003). The lack-of-fit was not significant (*p* = 0.2274 > 0.05), indicating that there was no significant evidence of model inadequacy and that the fitted model adequately described the experimental data. The coefficient of determination (*R*^2^) was 0.9875, indicating that the model accounted for 98.75% of the variation in the response. The adjusted coefficient of determination (adjusted *R*^2^) was 0.9651, which remained high after accounting for the number of model terms, further supporting the goodness of fit of the model. The F-values and corresponding *p*-values of the model terms were used to evaluate the significance of the effects of the independent variables on the response. The coefficient of variation (CV) was 2.13%, indicating good precision and reproducibility of the experimental results. Overall, these statistical parameters demonstrated satisfactory model adequacy and predictive performance, supporting the use of the fitted response surface model for the analysis, optimization, and prediction of total alkaloid extraction yield. Model adequacy was further evaluated using residual diagnostics and a predicted-versus-experimental plot (Figure S2).

Based on the *F*-value analysis, the influence of each factor on the extraction of total alkaloid content followed the order: *C* > *A* > *B*. Interactions represented the combined interaction effects of two factors acting together on the response value. The AB interaction reached a statistical significance level (0.01 < *p* < 0.05), while the BC interaction achieved a higher statistical significance level (*p* < 0.01). This indicated that considering the influence of individual factors alone is insufficient, and the interactions between variables must also be taken into account. The predicted optimal extraction conditions were: extraction time of 52.37 min, extraction temperature of 42.85 °C, and liquid-to-solid ratio of 39.12:1 (mL∙g^−1^).

### 2.4. Confirmatory Experiment of RSM

To validate the effectiveness of the response surface results, three parallel experiments were conducted using the optimized extraction conditions. For practical convenience, the extraction conditions were adjusted to: extraction time of 52 min, extraction temperature of 43 °C, and L/S ratio of 39:1 (mL∙g^−1^). Under the modified conditions, the TA yields obtained from the three parallel experiments were 291.207 mg∙g^−1^, 269.234 mg∙g^−1^, and 273.451 mg∙g^−1^, respectively, with a mean value of 277.964 ± 11.661 mg∙g^−1^ and a relative standard deviation (RSD) of 4.20%. The relative error of each replicate was calculated as the absolute difference between the experimental and model-predicted values divided by the model-predicted value and multiplied by 100%. The individual relative errors were 6.48%, 1.55%, and 0.01%, respectively, giving an average relative error of 2.68 ± 3.38%. These results indicate satisfactory agreement between the experimental and predicted values and support the reliability of the fitted model.

Previous studies have likewise reported improved alkaloid extraction performance using DES-based systems relative to conventional solvents. For example, He et al. reported that DES-based ultrasound-assisted extraction of total alkaloids from *Stephania tetrandra* achieved extraction efficiencies that were 2.2, 3.3, and 4.1 times those obtained using methanol, 95% ethanol, and water, respectively [29]. Yu et al. reported that a DES-based biphasic system increased the total alkaloid yield from *Evodia lepta* residue by 587.45% compared with conventional methanol extraction [13]. These quantitative improvements support the effectiveness of DES-based systems for plant alkaloid extraction. However, direct comparison of absolute alkaloid yields with the value obtained in the present study is not appropriate because endogenous alkaloid abundance and composition, plant matrix characteristics, extraction conditions, and analytical methods differ substantially among plant species and studies. Therefore, the present extraction yield should primarily be interpreted in the context of *E. lindleyanum* rather than as a direct indication of superiority over other plant systems.

### 2.5. Microstructure Characteristics and Antioxidant Activity of UAE-DES Extraction Products

**Figure 4:**
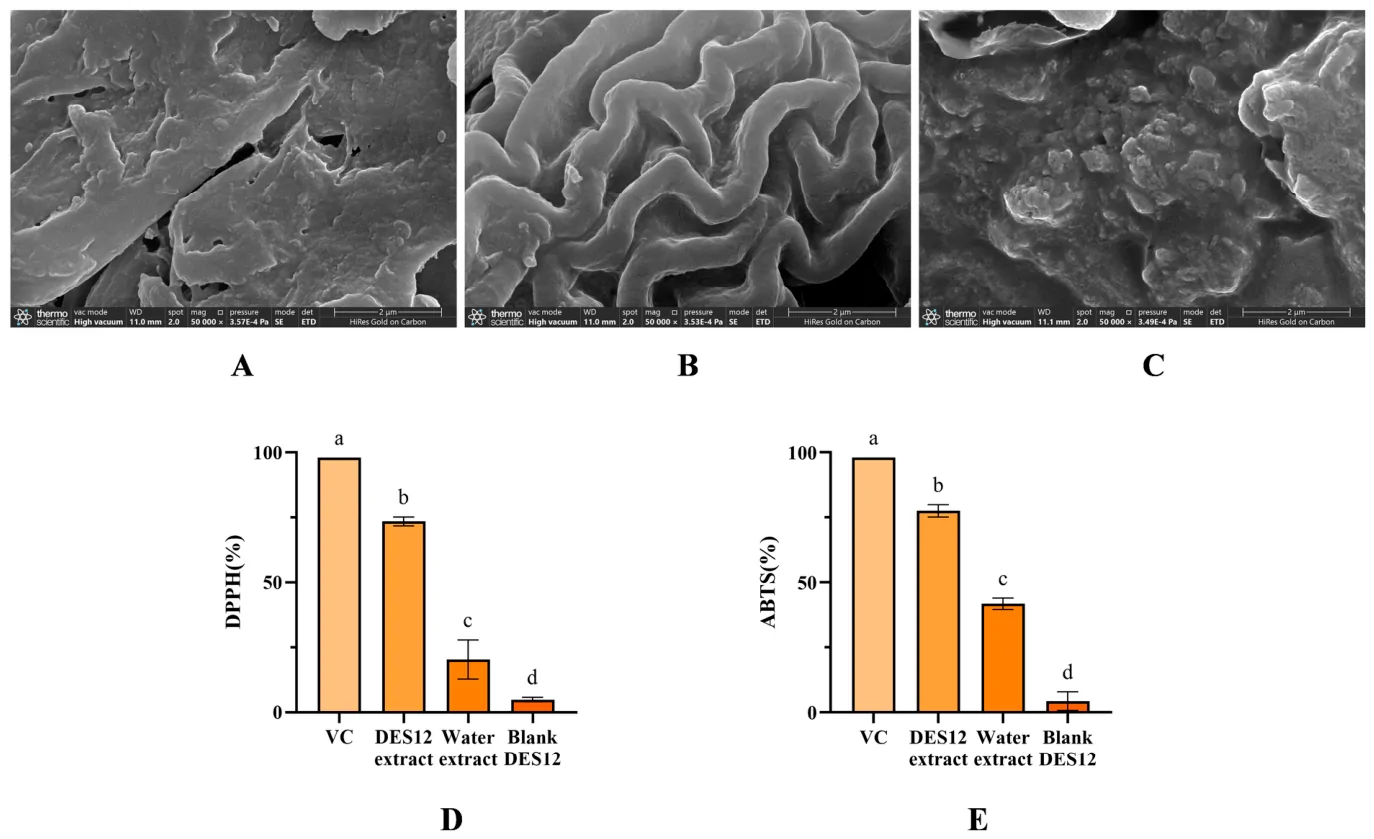
Microstructural changes in *E. lindleyanum* samples and antioxidant activity of different extracts. (**A**) Untreated sample; (**B**) sample after ultrasound-assisted water extraction; (**C**) sample after UAE-DES12 extraction; (**D**) DPPH radical scavenging activity of VC, DES12 extract, water extract, and blank DES12; (**E**) ABTS radical scavenging activity of VC, DES12 extract, water extract, and blank DES12. Data are expressed as mean ± SD (*n* = 3). Different lowercase letters indicate significant differences among groups according to one-way ANOVA followed by Tukey’s multiple comparison test (*p* < 0.05).

Enhanced extraction performance is closely associated with the disruption of plant cellular fibrous structures and the subsequent release of intracellular compounds. To examine the structural changes associated with UAE-DES extraction of *E. lindleyanum*, SEM was used to observe the microstructural changes in *E. lindleyanum* samples before and after extraction. The structural changes in *E. lindleyanum* samples after water extraction (50 °C, 50 min, liquid-to-solid ratio of 20 mL∙g^−1^) were also examined for comparison. Before extraction, the sample surface was relatively rough and exhibited large sheet-like structures (Figure 4A). After water extraction, obvious fibrous wrinkles appeared in the *E. lindleyanum* powder (Figure 4B), which may be attributed to the swelling and partial loosening of *E. lindleyanum* tissues during ultrasound-assisted water extraction. However, these structural changes were relatively limited, suggesting that only limited structural disruption occurred under the ultrasound-assisted water extraction conditions used in this study. In contrast, the cellular fibrous structure of *E. lindleyanum* was markedly disrupted after UAE-DES12 treatment (Figure 4C). Compared with ultrasound-assisted water extraction, UAE-DES12 treatment resulted in more pronounced structural modification of the *E. lindleyanum* tissues, which was consistent with its higher total alkaloid yield. The more pronounced structural modification observed after UAE-DES12 treatment may facilitate solvent penetration and the release of intracellular compounds. However, because a DES extraction group without ultrasound was not included, the independent and interactive contributions of DES and ultrasound cannot be distinguished from the present experimental design. Thus, the SEM observations provide qualitative morphological evidence of structural differences among the extraction treatments. Quantitative characterization of structural parameters such as pore size, porosity, and surface area would be required to further substantiate these morphological changes.

Antioxidant capacity is commonly used as an important indicator for evaluating the enrichment of bioactive constituents and the functional value of plant extracts. For instance, Li et al. demonstrated this by evaluating the antioxidant activity of alkaloid extracts from *Phellodendri amurensis* cortex using DES aqueous solutions [14]. Consistent with this notion, we further evaluated the antioxidant capacity of our extracts to compare their radical scavenging activities. As shown in Figure 4D,E, while VC served as a positive control with nearly 100% scavenging rates, the DES12 extract exhibited significantly enhanced performance compared to the water extract. Specifically, the scavenging rates against DPPH and ABTS radicals reached 72.74% and 77.35%, respectively, a substantial increase from the corresponding values of 20.56% and 41.59% for the water extract (*p* < 0.0001). The blank DES12 exhibited only limited DPPH and ABTS radical scavenging activity and was significantly lower than the DES12 extract (*p* < 0.05), suggesting that the higher radical-scavenging activity of the DES12 extract cannot be explained by the DES matrix alone. Because the DES12 and water extracts were evaluated at fixed concentrations rather than across concentration gradients, IC_50_ values were not calculated for these extracts. Concentration–response analysis of VC, used as the positive control, yielded IC50 values of 0.0119 mg∙mL^−1^ (95% CI: 0.01154–0.01223 mg∙mL^−1^) in the DPPH radical scavenging assay and 0.0168 mg∙mL^−1^ (95% CI: 0.01648–0.01709 mg∙mL^−1^) in the ABTS radical scavenging assay (Figure S3). These results indicate that, under the assay conditions used, the DES12 extract exhibited higher DPPH and ABTS radical scavenging activities than the water extract. Together with its higher total alkaloid yield, these findings suggest that UAE-DES12 extraction produced an *E. lindleyanum* extract with greater radical scavenging activity than that obtained by water extraction. Related UAE-DES studies have also reported substantial antioxidant activity. For example, Wang et al. reported DPPH and ABTS radical-scavenging rates above 90% for rosmarinic acid in a UAE-DES study of *Anchusa italica* flowers [19]. Although direct numerical comparison is limited by differences in sample composition, concentration, and assay conditions, these findings further support the potential of UAE-DES systems for obtaining plant-derived products with considerable radical-scavenging activity. Therefore, DES12 can be considered an effective and green extraction medium for obtaining *E. lindleyanum* extracts with high total alkaloid content and antioxidant activity, with potential application value in food preservation.

### 2.6. Effect of DES12 Extract on Postharvest Preservation of Grapes

In vitro antioxidant assays confirmed that the DES12 extract possessed strong free radical scavenging capacity and considerable potential as a natural bioactive agent. Building upon previous studies reporting the preservative potential of *E. lindleyanum* extracts for food applications [30], this study further investigated the postharvest preservation effect of DES12 extract on grapes to verify its practical application value.

**Figure 5:**
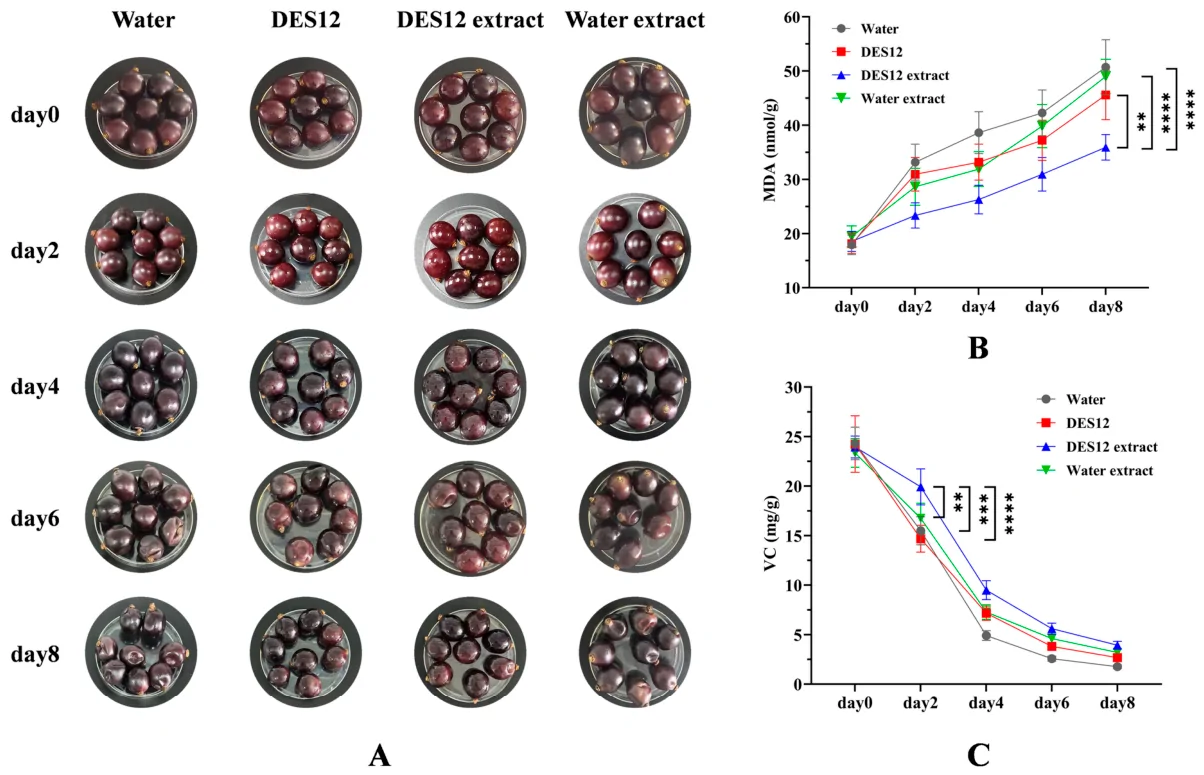
Effects of different treatments on the quality of postharvest grapes during storage. (**A**) Visual appearance of grapes during storage; (**B**) MDA content; (**C**) VC content. In the figure, “DES12” denotes the DES12 treatment without *E. lindleyanum* extract. Data in (**B**,**C**) are expressed as mean ± SD (*n* = 3). Statistical differences were analyzed using ordinary two-way ANOVA followed by Tukey’s multiple comparison test. For clarity, only significant differences among treatments at day 8 in (**B**) and at day 2 in (**C**) are indicated. Asterisks denote significant differences: ** *p* < 0.01, *** *p* < 0.001, and **** *p* < 0.0001.

The visual appearance of grapes during storage is presented in Figure 5A. All samples exhibited gradual quality deterioration over time; however, the degree of senescence differed among treatments. Compared with the water control, DES12, and water extract treatments, grapes treated with DES12 extract maintained better morphological integrity, with less surface shriveling and deterioration. By day 8, the water control grapes displayed obvious senescence and severe quality loss, whereas those in the DES12 extract group retained relatively intact fruit morphology, indicating better visual quality retention during storage.

MDA is a major by-product of membrane lipid peroxidation and is commonly used to evaluate oxidative stress-induced membrane damage in postharvest grapes [31]. Therefore, MDA content is an important indicator for evaluating oxidative deterioration and preservation performance during grape storage. As illustrated in Figure 5B, MDA content increased continuously across all groups throughout storage, indicating progressive oxidative deterioration. Ordinary two-way ANOVA showed significant effects of treatment (*p* < 0.0001), storage time (*p* < 0.0001), and their interaction (*p* = 0.0348) on MDA content. On day 8, the DES12 extract group exhibited the lowest MDA content, while the water control group showed the highest level, with significant differences observed between the DES12 extract group and the other treatment groups. These results were consistent with the visual appearance changes, further suggesting that DES12 extract may contribute to the maintenance of grape quality during storage. A similar relationship between reduced lipid peroxidation and improved grape storage quality has been reported in previous preservation studies. For example, Zeng et al. found that, after 60 days of storage, calcium-treated grapes had an MDA content of 19.61 μmol·kg^−1^ compared with 29.20 μmol·kg^−1^ in the control, corresponding to an approximately 32.8% reduction [32]. Although the treatment conditions and storage duration differed from those of the present study, the consistent suppression of MDA accumulation supports the relevance of limiting oxidative membrane damage for maintaining postharvest grape quality.

VC, a major non-enzymatic antioxidant, is closely correlated with the nutritional quality and endogenous antioxidant capacity of fruits [33]. As shown in Figure 5C, VC content decreased gradually in all samples due to the continuous consumption of antioxidants during senescence. Ordinary two-way ANOVA revealed significant effects of treatment (*p* < 0.0001), storage time (*p* < 0.0001), and their interaction (*p* = 0.0138) on VC content. The DES12 extract group showed better VC retention during storage, with significant differences from the water control observed at days 2, 4, and 6. At day 8, although the VC content remained numerically higher in the DES12 extract group, no significant differences were detected among treatments. Consistent with this trend, Zeng et al. reported that calcium-treated grapes retained 214.3 mg·kg^−1^ VC after 60 days of storage, approximately 2.32 times the level in the untreated control (92.3 mg·kg^−1^) [32]. Overall, the lower MDA accumulation and better VC retention observed in the DES12 extract group suggest that DES12 extract may alleviate oxidative deterioration and contribute to maintaining the nutritional quality of postharvest grapes during storage.

### 2.7. Protective Effect of DES12 Extract Against Gray Mold in Grapes

**Figure 6:**
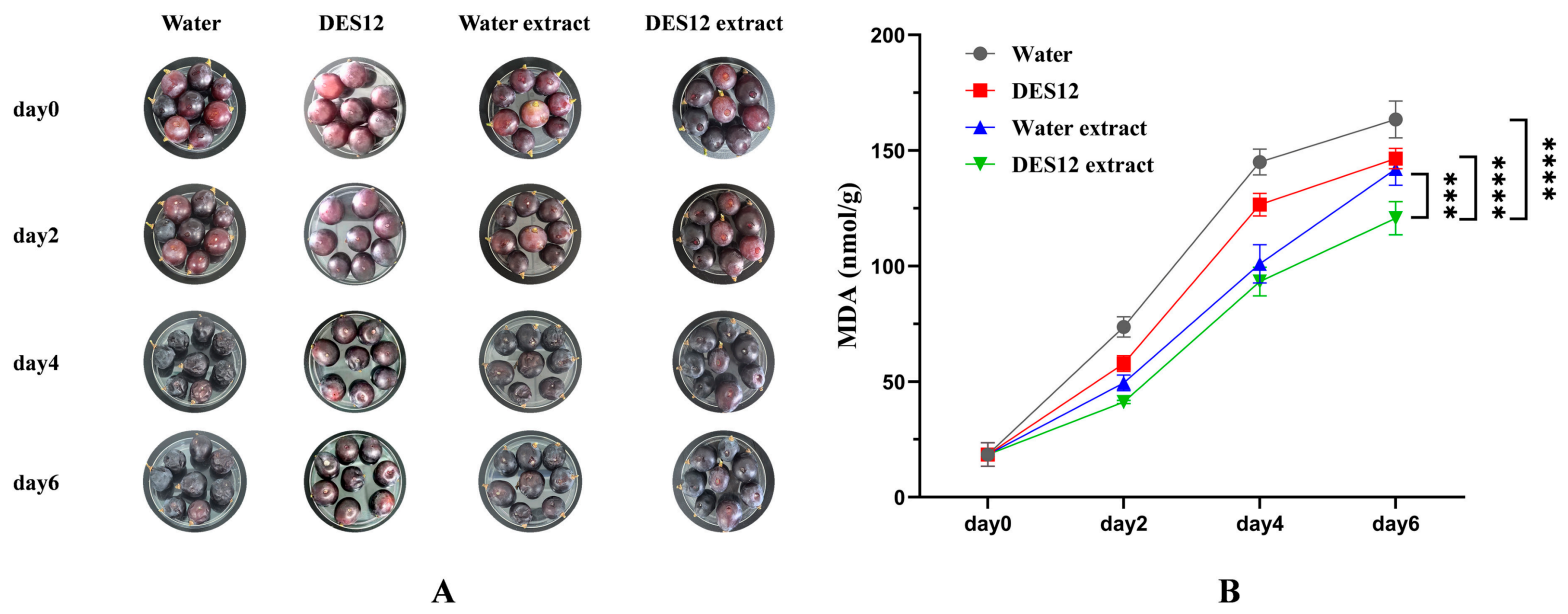
Protective effect of DES extract against *B. cinerea*-induced gray mold in grapes. (**A**) Visual appearance of infected grapes during storage; (**B**) MDA content in grapes after *B. cinerea* infection. In the figure, “DES12” denotes the DES12 treatment without *E. lindleyanum* extract. Data are expressed as mean ± SD (*n* = 3). Statistical differences were analyzed using ordinary two-way ANOVA followed by Tukey’s multiple comparison test. For clarity, only significant differences between the DES12 extract group and the other treatment groups at day 6 are indicated. Asterisks denote significant differences: *** *p* < 0.001 and **** *p* < 0.0001.

The aforementioned experiments preliminarily demonstrated that the *E. lindleyanum* DES12 extract showed potential for application in the postharvest preservation of grapes. Since *Botrytis cinerea* is a key causative agent of postharvest grape decay [34], we further evaluated the protective effect of the DES12 extract in a *B. cinerea*-infected grape model. As shown in Figure 6A, visible differences in gray mold-associated deterioration emerged among treatment groups during storage. At the beginning of storage, no apparent differences were observed among the groups. However, as storage progressed, grapes in the water control group exhibited obvious symptoms of decay, including tissue softening, discoloration, and shrinkage. In contrast, grapes treated with water extract showed less pronounced visible deterioration, while those treated with DES12 extract generally maintained a better appearance throughout the storage period. The DES12 group also showed some improvement in visual appearance compared with the water control, suggesting that the DES matrix itself may contribute to the observed protective effect. By day 6, the DES12 extract-treated grapes exhibited less severe visible deterioration than the water control, indicating a protective effect against *B. cinerea*-associated deterioration.

The changes in MDA content during storage are shown in Figure 6B. MDA is a widely recognized indicator of membrane lipid peroxidation and oxidative stress in plant tissues [35]. The MDA content increased continuously in all groups throughout storage, indicating progressive oxidative damage during *B. cinerea* infection. However, the increase in MDA content was markedly suppressed in the DES12 extract-treated grapes. The average MDA content of the water control group increased from 18.47 ± 5.12 nmol·g^−1^ on day 0 to 163.47 ± 8.00 nmol·g^−1^ on day 6. In comparison, the MDA contents of the DES12, water extract, and DES12 extract groups reached 146.54 ± 4.37, 142.03 ± 7.13 and 120.68 ± 7.26 nmol·g^−1^, respectively. Notably, the DES12 extract treatment reduced MDA accumulation by approximately 26.2% relative to the water control on day 6. Ordinary two-way ANOVA showed significant effects of treatment, storage time, and their interaction on MDA content (all *p* < 0.0001). Importantly, the DES12 treatment also reduced MDA accumulation relative to the water control at several storage times, indicating that the DES matrix itself may influence the physiological response of infected grapes. Nevertheless, MDA levels in the DES12 extract group were significantly lower than those in the DES12 group at days 2, 4, and 6 (*p* = 0.0046, *p* < 0.0001, and *p* < 0.0001, respectively), and were also significantly lower than those in the water extract group at day 6 (*p* = 0.0003).

The lower accumulation of MDA suggests that DES12 extract alleviated membrane lipid peroxidation and oxidative damage associated with *B. cinerea* infection. This protective effect may be associated with antioxidant constituents in the extract, which may contribute to ROS scavenging, reduction in oxidative stress, and preservation of membrane integrity. Similar findings have been reported in grapes, where enhancement of antioxidant defense systems and reduction in MDA accumulation were closely associated with improved resistance to *B. cinerea* infection [36]. In addition, previous studies on *E. lindleyanum* have shown that this medicinal plant contains diverse bioactive constituents, including alkaloids, flavonoids, sesquiterpenoids, and volatile oils, and exhibits multiple pharmacological activities, particularly antioxidant and antimicrobial effects. *E. lindleyanum* extracts have also been reported to inhibit food spoilage and foodborne pathogens, further supporting their potential role in microbial control [30]. Therefore, the protective effect observed in the present gray mold model may be related to the antioxidant capacity of the DES12 extract, while a possible contribution from the antimicrobial potential of *E. lindleyanum*-derived constituents cannot be excluded. Moreover, because the DES12 treatment also reduced MDA accumulation relative to the water control, the DES matrix itself may contribute to the overall protective response. However, the lower MDA content observed in the DES12 extract group compared with the DES12 group suggests an additional contribution from *E. lindleyanum*-derived constituents. These findings are consistent with the antioxidant capacity observed in the DPPH and ABTS assays, suggesting that antioxidant protection may contribute to the reduced oxidative damage observed in infected grapes.

However, the present results do not provide direct evidence of antifungal activity against *B. cinerea*. Because MIC, mycelial growth, spore germination, fungal biomass, lesion size, and disease incidence or severity were not quantitatively determined, direct inhibition of fungal growth cannot be distinguished from indirect protection of grape tissues through antioxidant or other physiological effects. Future studies incorporating direct antifungal assays and quantitative disease assessments will be required to clarify these mechanisms. Therefore, DES12 extract reduced visible gray mold-associated deterioration, limited MDA accumulation, and may have contributed to the preservation of tissue integrity during storage. Collectively, these results suggest that DES12 extract has considerable potential as a natural preservation agent for postharvest grapes, and its preservation effect may be closely associated with antioxidant protection, although a direct antifungal contribution remains to be established.

## 3. Materials and Methods

### 3.1. Reagents and Materials

*E. lindleyanum* was collected from Xiangkang Herbal Park (Huai’an, Jiangsu, China), dried, ground using a grinder, passed through a 60-mesh sieve, and stored in a dry place prior to use.

Betaine, choline chloride, lactic acid, decanoic acid, oleic acid, citric acid, xylitol, glycerol, malic acid, acetamide, ethylene glycol, thymol, menthol, vanillin, 2,2-diphenyl-1-picrylhydrazyl (DPPH), and 2,2′-azinobis(3-ethylbenzothiazoline-6-sulfonic acid) diammonium salt (ABTS) were obtained from Shanghai Yuanye Bio-Technology Co., Ltd. (Shanghai, China). Glucose was purchased from Tianjin Kemiou Chemical Reagent Co., Ltd. (Tianjin, China). Sucrose and dichloromethane were obtained from Sinopharm Chemical Reagent Co., Ltd. (Shanghai, China), and 1,4-butanediol was purchased from Shanghai Aladdin Bio-Chem Technology Co., Ltd. (Shanghai, China). All chemicals used in this study were of analytical grade unless otherwise specified.

The total alkaloid assay kit was purchased from Suzhou Grace Biotechnology Co., Ltd. (Suzhou, Jiangsu, China). The Plant Malondialdehyde (MDA) Assay Kit and Vitamin C (VC) Assay Kit were purchased from Nanjing Jiancheng Bioengineering Institute (Nanjing, China).

### 3.2. Preparation of DESs

DES was prepared by the heating method. According to the molar ratios listed in Table 4, the HBA and HBD were accurately weighed and mixed in a round-bottom flask. The mixture was heated and stirred in a water bath at 80 °C until completely dissolved to form a clear and transparent liquid. Subsequently, deionized water was added during stirring to adjust the water content to 30%. Hydrophobic DESs (DES16-18) did not require the addition of deionized water. After cooling, the DES was sealed and stored in a dry container.

### 3.3. Determination of Physicochemical Properties of DESs

The density and polarity of DES1, DES6, DES7, and DES12 were determined at 25 ± 1 °C. Density was measured using the equal-volume weighing method. Briefly, a clean and dry weighing bottle was weighed (*m*_0_), filled with distilled water to a fixed volume and weighed again (*m*_1_), and then filled with the same volume of DES and weighed (*m*_2_). The density of the DES was calculated according to Equation (1):

(1) $$\rho_{\mathrm{DES}}\;=\;\frac{m_{2}\;-\;m_{0}}{m_{1}\;-\;m_{0}}\;\times \;\rho_{\mathrm{water}}$$

where *ρ_water_* is the density of water at the measurement temperature.

The polarity of DES1, DES6, DES7, and DES12 was determined using Nile red as a solvatochromic probe according to a previously reported method with slight modifications [21]. Briefly, Nile red was dissolved in 96% (*v*/*v*) ethanol to prepare a 5 mM stock solution. Subsequently, 20 µL of the Nile red solution was mixed with 980 µL of each DES sample and vortexed thoroughly. The maximum absorption wavelength (*λ*_max_) of the resulting solution was recorded over the range of 400–700 nm using a UV–Vis spectrophotometer (UV-2401PC, Shimadzu Corporation, Kyoto, Japan). The polarity parameter (*E*_NR_) was calculated according to Equation (2):

(2) $$E_{\mathrm{NR}}=\frac{28591.44}{\lambda_{\max}}$$

Density measurements were performed in triplicate, and the results are expressed as mean ± SD. The polarity parameter (*E_NR_*) was calculated from the experimentally determined *λ_max_* of Nile red and is reported as a calculated physicochemical parameter.

### 3.4. Extraction Process

The powdered *E. lindleyanum* was mixed with the prepared DES at a liquid-to-solid ratio of 20:1 mL∙g^−1^, followed by ultrasonic extraction at 50 °C for 50 min. After ultrasonication, the mixture was transferred to a centrifuge tube and centrifuged at 5000 r∙min^−1^ for 10 min. The supernatant was collected, and 40 μL of the supernatant was used for the total alkaloid assay. The TA yield (mg∙g^−1^) was used as an indicator for screening the appropriate solvent.

### 3.5. Determination of Total Alkaloid Yield

The total alkaloid yield was determined using the total alkaloid assay kit provided by Suzhou Grace Biotechnology Co., Ltd. (Suzhou, Jiangsu, China). Briefly, 0.6 mL of Reagent B was added to Solution A, thoroughly mixed until completely dissolved, and then transferred entirely to Reagent 2. The mixture was vortexed to prepare the working solution for subsequent use. For sample analysis, 40 μL of the supernatant obtained from centrifugation was mixed sequentially with 400 μL of dichloromethane, 200 μL of distilled water, and 80 μL of the working solution. The blank group was prepared without adding the sample. The mixture was manually shaken up and down for 5 min and then allowed to stand at room temperature (25 °C) for 30 min. Subsequently, 200 μL of the clear lower layer was carefully transferred to a 96-well UV plate, and the absorbance was promptly measured at 415 nm. A calibration curve was constructed using the reference standard supplied with the commercial assay kit. The specific chemical identity of the reference standard was not disclosed by the manufacturer. Standard solutions were prepared at concentrations of 0, 40, 120, 160, and 200 μg·mL^−1^. The absorbance difference at 415 nm (ΔOD_415_ = OD_sample_ − OD_blank_) was used for linear regression, yielding the calibration equation *y* = 0.0038*x* − 0.0148 (*R*^2^ = 0.9927) over the calibration range of 0–200 μg·mL^−1^ (Figure S4), where x represents the standard concentration and y represents ΔOD_415_. The total alkaloid content was calculated from the calibration equation and expressed as mg·g^−1^ of dry *E. lindleyanum* material. Because the assay is colorimetric and non-compound-specific, its selectivity in the DES matrix was not independently validated in the present study. In addition, no HPLC or LC–MS characterization was performed; therefore, the reported TA values should be interpreted as total alkaloid estimates obtained using the commercial colorimetric assay rather than as compound-specific alkaloid concentrations.

### 3.6. Single-Factor Experiment

Time (30–70 min), temperature (30–70 °C), and liquid-to-solid ratio (10:1–50:1 mL∙g^−1^) were used as the influencing factors to investigate their effects on the total alkaloid extraction yield and to conduct the next step of response surface methodology.

### 3.7. Response Surface Method (RSM)

The experimental design was conducted using Box–Behnken design (BBD), and response surface methodology (RSM) was performed using Design-Expert software version 13 (Stat-Ease, Inc., Minneapolis, MN, USA) to explore the optimal extraction process. Through modeling and data analysis, the influencing factors on the total alkaloid content were optimized. A total of 15 experimental runs were performed in a randomized order. Each experimental condition was performed in triplicate, and the response values are expressed as mean ± SD. Among these, three main factors were selected as independent variables, including extraction time (*A*: 40–60 min), extraction temperature (*B*: 40–60 °C), and liquid-to-solid ratio (*C*: 20:1–40:1 mL∙g^−1^), with the total alkaloid extraction yield (*Y*) as the response variable.

### 3.8. Scanning Electron Microscopy (SEM) Analysis

The *E. lindleyanum* morphology was analyzed using a field emission scanning electron microscope (Quattro S, Thermo Fisher Scientific, Waltham, MA, USA) to compare the morphological and structural characteristics of *E. lindleyanum* samples subjected to different extraction treatments. Approximately 10 mg of each sample was oven-dried and then attached to conductive adhesive for testing. The prepared samples were then placed in the SEM chamber and scanned using an electron beam to capture topographical details. Imaging was conducted at a magnification of 50,000×.

### 3.9. Antioxidant Activity Test

The antioxidant activity of the extracts was evaluated using DPPH and ABTS radical scavenging assays. For the DPPH assay, 1 mL of extract was mixed with 2 mL of DPPH ethanol solution (0.2 mM), incubated in the dark at room temperature for 30 min, and the absorbance was measured at 517 nm. For the ABTS assay, ABTS radical cation solution was prepared by reacting ABTS solution (7.4 mmol∙L^−1^) with K_2_S_2_O_8_ solution (2.6 mmol∙L^−1^) in the dark for 12 h and diluted with ethanol before use. Then, 0.6 mL of sample solution was mixed with 1.6 mL of ABTS solution, and the absorbance was measured at 734 nm. Vitamin C (VC, 2 mg∙mL^−1^) was used as a positive control. Blank DES12 was evaluated in parallel to assess the radical scavenging activity of the DES matrix. In addition, concentration–response assays were performed with VC for IC_50_ determination. The IC_50_ value, defined as the VC concentration required to achieve 50% radical scavenging activity, was determined from the concentration–response curve using nonlinear regression in GraphPad Prism 10. All measurements were performed in triplicate, and radical scavenging activity was calculated according to Equation (3):

(3) $$\mathrm{Radical}\;\mathrm{scavenging}\;\mathrm{activity}\;(\%)\;=\;\frac{A_{0}\;-{\;A}_{s}}{A_{0}}\;\times \;100$$

where *A*_0_ is the absorbance of the blank and *A*_s_ is the absorbance of the sample.

### 3.10. Grape Preservation Test

Fresh ‘Kyoho’ grapes with uniform maturity (approximately 8 °Brix), free from mechanical damage and visible disease symptoms, were selected as experimental materials. The grape berries were surface-sterilized with 70% ethanol for 30 s and randomly divided into four groups (30 berries per group): water control, DES12, DES12 extract, and water extract. The water control group was treated with distilled water, whereas the DES12 group was treated with DES12 without *E. lindleyanum* extraction. The DES12 extract group was treated with the DES12 extract prepared under the optimized UAE-DES conditions (52 min, 42 °C, and a liquid-to-solid ratio of 39:1 mL·g^−1^), which contained 0.28 mg·mL^−1^ total alkaloids. The water extract was prepared at 50 °C for 50 min using a liquid-to-solid ratio of 20:1 mL·g^−1^. Both extracts were used directly in liquid form without isolation of the dried crude extracts. Each grape berry was uniformly coated with the corresponding treatment solution using a soft brush and stored at 25 ± 2 °C for 8 days.

At each sampling time (0, 2, 4, 6, and 8 days), three individual berries were randomly selected from each treatment group for destructive sampling and biochemical analysis. Each berry was regarded as one experimental unit; therefore, *n* = 3 for each treatment at each sampling time. Thus, 15 berries per treatment were used for MDA and VC measurements, while the remaining 15 berries were retained for visual observation during storage and as reserve samples.

During storage, MDA and VC contents were determined every 48 h using commercial assay kits according to the manufacturer’s instructions. MDA content was measured using a Plant Malondialdehyde Assay Kit, and VC content was determined using a Vitamin C Assay Kit (Nanjing Jiancheng Bioengineering Institute, Nanjing, China). Fresh grape tissue was homogenized under ice-bath conditions and centrifuged to obtain the supernatant for analysis. The absorbance values for MDA and VC were measured at 530 and 536 nm, respectively. MDA and VC contents were expressed as nmol∙g^−1^ and mg∙g^−1^ fresh weight, respectively.

### 3.11. Gray Mold Infection and Preservation Test

Fresh ‘Kyoho’ grapes with uniform maturity and free from visible defects were selected. The grape berries were randomly divided into four groups, with 30 berries per group. Each berry was inoculated with 10 μL of *B. cinerea* spore suspension (1 × 10^5^ spores∙mL^−1^) using a microsyringe. Subsequently, the berries were injected with 20 μL of sterile water (Water), DES12 without *E. lindleyanum* extract (DES12), water extract (Water extract), or DES12 extract (DES12 extract), respectively.

After treatment, all grape berries were stored at 25 ± 2 °C under ambient relative humidity for up to 6 days, and observations were recorded on Days 0, 2, 4, and 6. During storage, MDA content was determined every 48 h using the commercial assay kit as described in Section 3.10. At each sampling time, three individual berries were randomly selected from each treatment group for destructive MDA analysis. Each berry was regarded as one experimental unit; therefore, *n* = 3 for each treatment at each sampling time.

### 3.12. Statistical Analysis

All data are expressed as mean ± SD. Statistical analyses were performed using GraphPad Prism 10 (GraphPad Software, Boston, MA, USA). For experiments involving a single factor, differences among groups were analyzed by one-way ANOVA followed by Tukey’s multiple comparison test or by the Kruskal–Wallis test followed by Dunn’s multiple comparison test, as appropriate. For the grape preservation and *B. cinerea* infection experiments, data involving treatment and storage time were analyzed using ordinary two-way ANOVA, with treatment and storage time as the two factors, including their interaction, followed by Tukey’s multiple comparison test for comparisons among treatments at each storage time. Figures were generated using GraphPad Prism 10, and *p* < 0.05 was considered statistically significant.

## 4. Conclusions

In this study, a green UAE-DES strategy was developed for the efficient extraction of total alkaloids from *E. lindleyanum*. Among the 18 DES systems evaluated, choline chloride–citric acid (2:1, molar ratio) was identified as the optimal solvent, and the extraction conditions were optimized to 52 min, 42 °C, and a liquid-to-solid ratio of 39:1 mL∙g^−1^, yielding 277.96 mg∙g^−1^ total alkaloids. SEM analysis showed pronounced structural disruption of *E. lindleyanum* tissues after UAE-DES treatment. The obtained DES extract exhibited strong antioxidant activity. In postharvest grape preservation, DES12 extract reduced MDA accumulation and maintained higher VC levels, thereby alleviating oxidative damage and delaying quality deterioration. In the *B. cinerea* infection model, DES12 extract reduced visible gray mold-associated deterioration and alleviated oxidative damage in inoculated grapes, suggesting its potential to mitigate gray mold-associated deterioration. These findings suggest that UAE-DES extraction not only improves the recovery of *E. lindleyanum* alkaloids but also provides an application-oriented strategy for developing plant-derived natural antioxidant-based preservation agents for postharvest fruit preservation. Nevertheless, further studies on process scale-up, production cost, extract stability, safety, and regulatory compliance are needed to support the translation of this approach from laboratory-scale validation to practical food preservation applications.

## Figures and Tables

**Table 1:** Composition and physicochemical properties of the selected DESs.

DES Code	HBD	HBA	Molar Ratio	Density (g∙cm^−3^)	*E*_NR_
DES1	d-glucose	Citric Acid	1:1	1.3384 ± 2 ×10^−4^	44.6
DES6	Betaine	Glucose	1:1	1.2999 ± 2 ×10^−4^	58.5
DES7	Lactic acid	Sucrose	1:1	1.3057 ± 1 ×10^−4^	47.7
DES12	Choline chloride	Citric Acid	2:1	1.3213 ± 2 ×10^−4^	44.7

**Table 2:** Experimental design and results of the Box–Behnken design. Data are expressed as mean ± SD (*n* = 3).

NO	*A*	*B*	*C*	*Y*
1	40	40	30	208.158 ± 3.93
2	60	40	30	223.043 ± 7.40
3	40	60	30	215.058 ± 8.17
4	60	60	30	204.704 ± 2.33
5	40	50	20	190.647 ± 8.70
6	60	50	20	196.852 ± 7.68
7	40	50	40	226.142 ± 3.84
8	60	50	40	251.908 ± 3.49
9	50	40	20	190.938 ± 5.30
10	50	60	20	219.682 ± 6.35
11	50	40	40	272.906 ± 3.58
12	50	60	40	233.519 ± 5.77
13	50	50	30	249.999 ± 7.49
14	50	50	30	251.259 ± 8.55
15	50	50	30	255.757 ± 8.23

**Table 3:** Box–Behnken test design analysis.

Source	Sum of Square	df	Mean Square		*F*-Value	*p*-Value	Significant
Model	9180.43	9	1020.05		43.98	0.0003	Significant
*A*	166.55	1	166.55		7.18	0.0438	
*B*	60.95	1	60.95		2.63	0.1659	
*C*	4341.07	1	4341.07		187.16	<0.0001	
*AB*	159.25	1	159.25		6.87	0.0471	
*AC*	95.66	1	95.66		4.12	0.0980	
*BC*	1160.46	1	1160.46		50.03	0.0009	
*A* ^2^	2541.48	1	2541.48		109.57	0.0001	
*B* ^2^	659.22	1	659.22		28.42	0.0031	
*C* ^2^	348.51	1	348.51		15.03	0.0117	
Residual	115.97	5	23.19				
Lack of fit	97.65	3	32.55		3.55	0.2274	Not significant
Pure Error	18.32	2	9.16				
Cor Total	9296.41	14					
*R*^2^ = 0.9875				Adjusted *R*^2^ = 0.9651			
Predicted *R*^2^ = 0.8275				Adeq Precision = 20.5107			

**Table 4:** DES prepared in this research.

	HBD	HBA	Molar Ratio
1	d-glucose	Citric Acid	1:1
2	Betaine	Citric Acid	1:2
3	Choline chloride	Xylitol	2:1
4	Choline chloride	Lactic acid	1:1
5	Choline chloride	Glycerol	1:2
6	Betaine	Glucose	1:1
7	Lactic acid	Sucrose	1:1
8	Choline chloride	Sucrose	1:1
9	Choline chloride	Glucose	1:1
10	Choline chloride	Malic acid	1.5:1
11	Choline chloride	Acetamide	1:2
12	Choline chloride	Citric Acid	2:1
13	Choline chloride	Citric Acid	1:2
14	Choline chloride	Ethylene glycol	1:2
15	Choline chloride	Butane-1,4-diol	1:2
16	Capric acid	5-methyl-2-Isopropylphenol	1:1
17	Oleic acid	5-methyl-2-Isopropylphenol	1:1
18	Oleic acid	l-Menthol	1:1

## Data Availability

The raw data supporting the conclusions of this study are available from the corresponding author upon reasonable request.

## Supplementary Materials

The following supporting information can be downloaded at: https://www.mdpi.com/article/10.3390/molecules31183352/s1, Figure S1, Correlations between the physicochemical properties of selected DESs and total alkaloid yield. (**A**) Correlation between density and TA yield; (**B**) correlation between E_NR and TA yield; Figure S2, Diagnostic plots for the fitted Box–Behnken response surface model. (**A**) Normal probability plot of externally studentized residuals; (**B**) externally studentized residuals versus predicted values; (**C**) predicted versus actual TA yields; Figure S3, Concentration–response curves of VC in antioxidant assays. (**A**) DPPH radical scavenging activity; (**B**) ABTS radical scavenging activity; Figure S4, Calibration curve for total alkaloid determination using the reference standard supplied with the commercial assay kit; Table S1, Detailed two-way ANOVA results for MDA and VC in the grape preservation experiment and MDA in the *Botrytis cinerea* infection experiment.
