# Supplementary material for: Ultrasound-Assisted Deep Eutectic Solvent Extraction of Total Alkaloids from Eupatorium lindleyanum: Process Optimization and Preservation Application in Postharvest Grapes
Source: Molecules. 2026 Sep 21;31(18):3352. doi: 10.3390/molecules31183352 (PMC13610118; doi:10.3390/molecules31183352)
Supplement: Supplementary file 1 [file molecules-31-03352-s001.zip › molecules-4523731-supplementary.pdf]

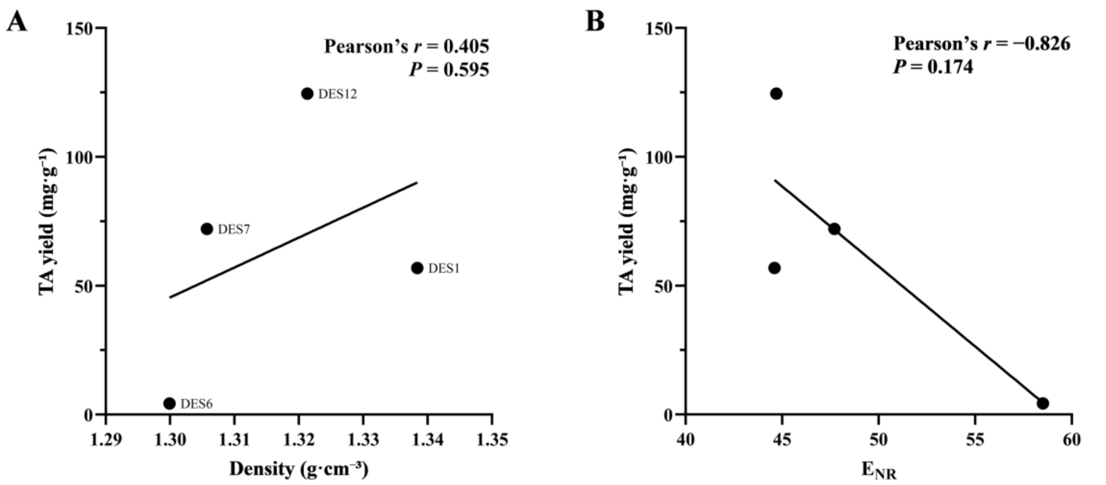

Figure S1. Correlations between the physicochemical properties of selected DESs and total alkaloid yield. (**A**) Correlation between density and TA yield; (**B**) correlation between E_NR and TA yield.

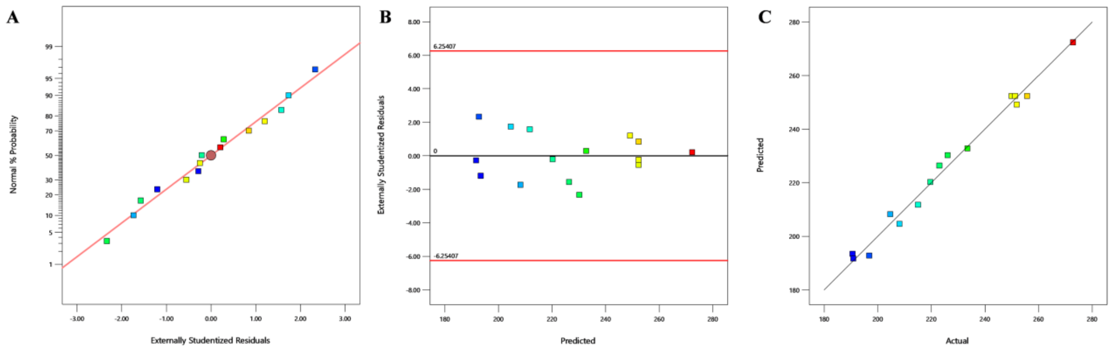

Figure S2. Diagnostic plots for the fitted Box–Behnken response surface model. (**A**) Normal probability plot of externally studentized residuals; (**B**) externally studentized residuals versus predicted values; (**C**) predicted versus actual TA yields.

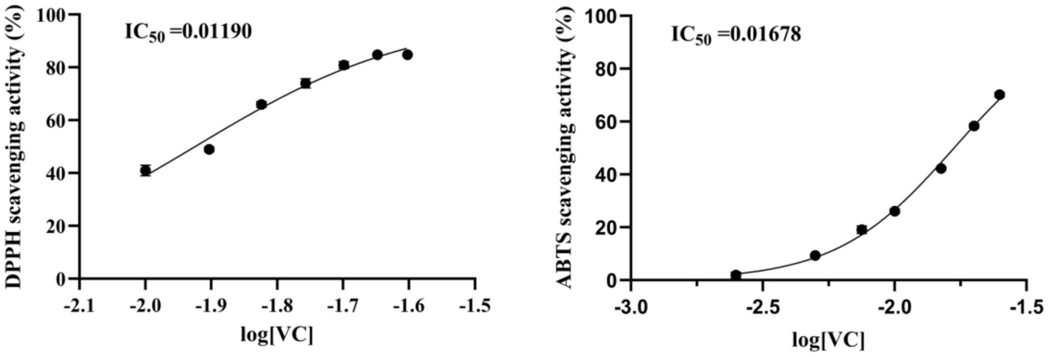

A **B**

Figure S3. Concentration–response curves of VC in antioxidant assays. (**A**) DPPH radical scavenging activity; (**B**) ABTS radical scavenging activity.

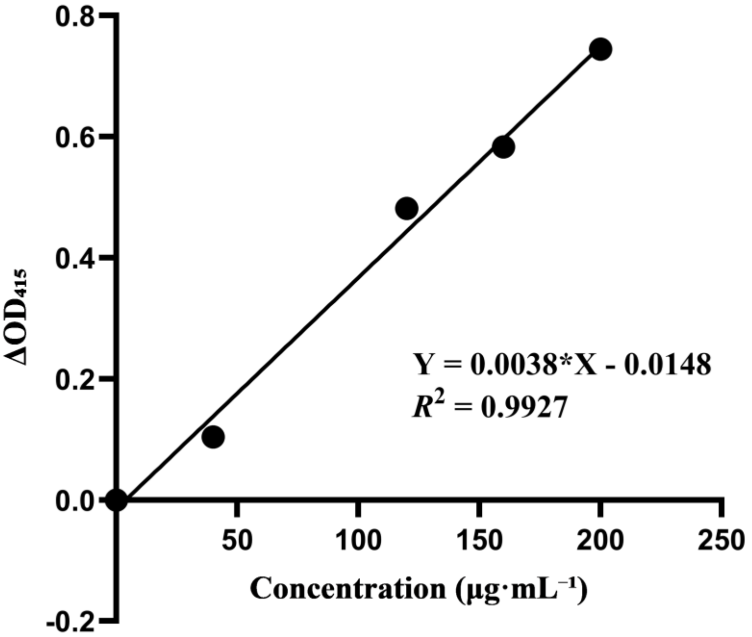

Figure S4. Calibration curve for total alkaloid determination using the reference standard supplied with the commercial assay kit.

Table S1. Detailed two-way ANOVA results for MDA and VC in the grape preservation experiment and MDA in the *Botrytis cinerea* infection experiment.

Experiment	Response variable	Source of variation	SS	df	MS	F (DFn, DFd)	*P* value
Grape preservation	MDA	Treatment	724.9	3	241.6	22.60 (3, 40)	<0.0001
		Storage time	4764	4	1191	111.4 (4, 40)	<0.0001
		Treatment × Storage time	276.2	12	23.02	2.153 (12, 40)	0.0348
		Residual	427.7	40	10.69	—	—
Grape preservation	VC	Treatment	62.31	3	20.77	15.42 (3, 40)	<0.0001
		Storage time	3932	4	982.9	729.5 (4, 40)	<0.0001
		Treatment × Storage time	40.94	12	3.411	2.532 (12, 40)	0.0138
		Residual	53.89	40	1.347	—	—
B. cinerea infection	MDA	Treatment	6651	3	2217	71.35 (3, 32)	<0.0001
		Storage time	115907	3	38636	1243 (3, 32)	<0.0001
		Treatment × Storage time	2968	9	329.8	10.61 (9, 32)	<0.0001
		Residual	994.3	32	31.07	—	—

Note: Ordinary two-way ANOVA was performed with treatment and storage time as the two factors, including their interaction. For the grape preservation experiment, four treatment groups and five storage times were included, with three independent biological replicates for each treatment at each sampling time (n = 3), giving a total of 60 observations for each response variable. For the *Botrytis cinerea* infection experiment, four treatment groups and four storage times were included, with three independent biological replicates for each treatment at each sampling time (n = 3), giving a total of 48 observations. SS, sum of squares; MS, mean square; DFn, numerator degrees of freedom; DFd, denominator degrees of freedom.
